# The Role of Post-Translational Modifications in the Phase Transitions of Intrinsically Disordered Proteins

**DOI:** 10.3390/ijms20215501

**Published:** 2019-11-05

**Authors:** Izzy Owen, Frank Shewmaker

**Affiliations:** Department of Biochemistry, Uniformed Services University of the Health Sciences, Bethesda, MD 20814, USA; izzy.owen@usuhs.edu

**Keywords:** liquid–liquid phase separation, intrinsically disordered regions, post-translational modifications, membraneless organelles

## Abstract

Advances in genomics and proteomics have revealed eukaryotic proteomes to be highly abundant in intrinsically disordered proteins that are susceptible to diverse post-translational modifications. Intrinsically disordered regions are critical to the liquid–liquid phase separation that facilitates specialized cellular functions. Here, we discuss how post-translational modifications of intrinsically disordered protein segments can regulate the molecular condensation of macromolecules into functional phase-separated complexes.

## 1. Introduction to Liquid–Liquid Phase Separation and Membraneless Organelles

Cells contain crowded molecular environments hosting discrete functions that must be separated within time and space. Membrane-less compartments resulting from liquid–liquid phase separation (LLPS) are increasingly being recognized as mechanisms for organizing cellular activities. These distinct regions may be referred to as biomolecular condensates or membrane-less organelles (MLOs). As the names suggest, these organelles are not encapsulated in a membrane, yet contain enriched sets of specific macromolecules. Thus, LLPS is the biologically regulated process by which specific macromolecular components are concentrated into a specific MLO.

MLOs contain proteins, and frequently nucleic acids, and are dynamic in size (generally submicrometer), formation, and composition [1]. They behave like liquid droplets, capable of fusing, deforming, and rearranging [2]—all while being solvated in the larger aqueous environment of the cell. The macromolecular components of MLOs have a higher affinity for each other than for surrounding molecules, allowing for separation from the bulk solution by demixing, thus forming two co-existing liquid states with differing concentrations of particular solutes [3].

The network of multivalent interactions within an MLO is not ordered like a conventional protein complex [4,5,6]. The interactions are typically characterized as non-static and more dynamic, with less specificity and weaker binding than the forces that hold macromolecular complexes—such as the proteasome or ribosome–into rigid stoichiometric structures [2]. For example, a ribosome consists of large and small subunits with more-or-less specific quaternary arrangement of components that together form a large macromolecular machine. Interactions in MLOs are thought to be less specific, with greater fluctuation of molecular contacts and stoichiometry. The plasticity of interactions may permit these organelles to react more dynamically to specific cellular conditions.

Numerous distinct functional MLOs have been characterized, and their many unique protein constituents have been previously reviewed [7,8]. A recently developed database of nearly 3000 non-redundant LLPS-associated proteins suggests that many MLOs have yet to be fully characterized [9]. Of the MLOs that have been characterized, their diversity and ubiquity is remarkable. MLOs have been observed in cytoplasm and nucleoplasm, and also in canonical membrane-enclosed organelles like mitochondria or chloroplasts [10]. Most commonly, MLOs are linked to specific functions involving ribonucleic acid, such as germ granules [11]. Pathological examples have also been proposed, such as the cytoplasmic inclusion bodies (IBs) within which measles viral RNA is replicated [12]. MLOs may exist transiently, like stress granules (SGs), which are stalled translation complexes that form upon cellular stress [13]. Alternatively, MLOs can have a more persistent presence, like the nucleolus, which is a constant site of ribosome production in the nucleus [7,14]. MLOs may also form in response to spatial necessity, such as neuronal RNA granules, which function in transport of mRNAs from dendrite bodies to distant synapses [15,16].

MLOs may also have roles in the pathogenesis of many diseases, particularly neurodegenerative disorders [17]. For example, many proteins linked to amyotrophic lateral sclerosis (ALS) and frontotemporal dementia (FTD) can undergo LLPS and accumulate within MLOs [18]. Mutations in these proteins not only cause disease but can alter LLPS and the physical properties of the phase-separated state [19,20]. It is hypothesized that aberrant irreversible phase transitions may result in proteinaceous neuronal inclusions that lead directly to cellular dysfunction [2].

## 2. Intrinsically Disordered Regions Facilitate LLPS

An interesting feature of proteins that undergo LLPS is they frequently contain long segments that lack well-defined three-dimensional structure. These segments are typically termed intrinsically disordered regions (IDRs), or intrinsically disordered proteins (IDPs), because they have no single equilibrium structure; instead, they exist as broad structural (or population) ensembles or they exchange between multiple conformations rapidly. IDRs are usually defined as being approximately 30 amino acids or longer [21], and their distinguishing characteristic is a relative paucity of hydrophobic amino acids to drive folding into a narrow conformational landscape.

The sequence composition of IDRs can vary, but is commonly disproportionately represented by only a few amino acids (i.e., low-complexity). Some low-complexity sequences are called yeast prion-like because they are compositionally very similar to the domains that enable certain yeast proteins to form self-propagating amyloid fibers. Yeast prion domains (and prion-like domains (PrLDs)) are usually very rich in hydrophilic amino acids (e.g., asparagine, glutamine, serine, and tyrosine). Other low-complexity sequences may disproportionately contain charged amino acids, such as the arginine/glycine repeats (RGG or GRG), which occur in several IDRs within liquid phase-separating proteins. Repeating (or spatially distributed) motifs of a subset of amino acids are also common to IDRs [6]. IDRs of MLO-forming proteins are also enriched in amino acids that can form π–π interactions, in which induced electrostatic interactions occur between sp^2^ hybridized atoms [22]. These interactions can also involve the backbone amide bonds, which are accessible due to the non-folded arrangement of IDRs.

The significance of IDRs in liquid phase-separating proteins is they enable diverse networks of transient interactions with moderate affinities (i.e., reversible, due partly to entropic penalties of IDRs adopting binding conformations). Relative to folded domains, IDRs have greater accessible conformational space and flexibility for forming molecular contacts. The frequent presence of repetitive motifs can enable numerous low-affinity interactions with the potential for high-avidity binding [23]. IDRs can therefore support multivalent interactions, meaning they can form multiple molecular contacts with a potential variety of binding partners. Thus, an MLO may emerge from a continuous network of IDRs forming inter-protein (or RNA-protein) multivalent contacts.

## 3. Post-Translational Modifications of Intrinsically Disordered Domains Can Govern LLPS

The lack of secondary structure makes IDRs especially susceptible to post-translational modifications (PTMs) [24]. In fact, IDRs are disproportionately modified post-translationally relative to the entire proteome [25,26,27]. A variety of PTMs can alter IDR charge, hydrophobicity, size, and structure. These changes may occur through additions of functional groups (e.g., phosphoryl, methyl, acyl, glycosyl, alkyl, etc.), or subtler chemical changes such as oxidation, deimidation, and deamidation [28].

**Figure 1:**
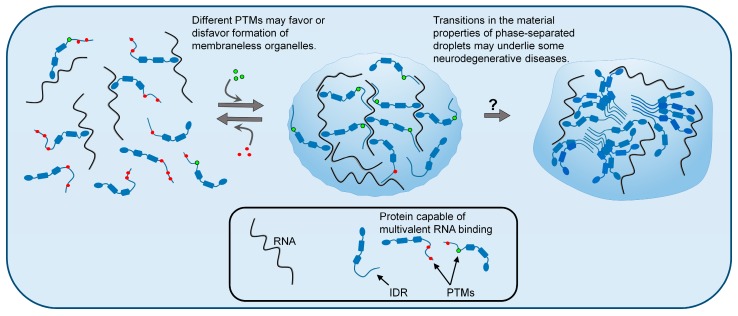
Liquid–liquid phase separation (LLPS) of biopolymers, such as proteins and RNA, is a mechanism by which cells organize their contents into specific functional structures called membraneless organelles (MLOs). Post-translational modifications (PTMs) of intrinsically disordered proteins can influence LLPS and thus regulate the formation and dissolution of MLOs. The figure depicts different patterns of PTMs favoring dispersed or condensed states. Changes in the material properties of liquid-phase separated granules are hypothesized to cause some neurodegenerative diseases. According to this hypothesis, droplets lose their liquid (reversible) properties and adopt more rigid (less reversible) internal structures, which may be glass-like, or in some cases, may have solid amyloid-like structures. These irreversible phase states may have gain-of-function toxicity to neurons.

There are many biological examples of PTMs serving as on/off switches, where they regulate a cellular event, such as protein signaling, localization, and degradation. In the case of IDRs and phase separation, PTMs can similarly have on/off functions by altering the nature of intermolecular contacts that support MLO formation or dissolution [29] (Table 1). Here, we discuss examples of PTMs and IDRs in proteins that undergo functional phase separation in cells. We evaluate the hypothesis that the combination of IDR multivalency and the capacity to be extensively modified results in reversible networks of interactions that can be regulated by specific cellular cues (Figure 1).

### 3.1. Serine/Threonine/Tyrosine Phosphorylation

Phosphorylation is the covalent attachment of a phosphoryl group to an amino acid hydroxyl group. The phosphoryl group is negatively charged, so its addition changes a polar, uncharged residue to a negatively charged amino acid. Serine is the most commonly phosphorylated residue, followed by threonine and tyrosine [30]. The addition of charges to macromolecules may promote certain charge–charge interactions that drive complex coacervation (phase separation of oppositely charged polymers) [31]. Alternatively, addition of phosphates may cause charge repulsion or steric hindrance, thus inhibiting phase separation [2]. Depending on the protein context, the phosphate modification of amino acids can either favor or disfavor phase separation.

Serine/threonine phosphorylation has been shown to promote phase separation of IDPs such as fragile X mental retardation protein (FMRP) [32], TIA-1/TIAL RNA binding protein homolog (TIAR-2) [33], and microtubule-binding protein tau [34]. FMRP has 12 serine residues within its C-terminal IDR (aa 445–632) that have been identified as targets of casein kinase II (CKII). In vitro phosphorylation by CKII results in an increase in the negative charge densities throughout this IDR, increasing the propensity for multivalent electrostatic interactions and promoting phase separation [32]. TIAR-2 also contains a C-terminal intrinsically disordered PrLD that facilitates its LLPS into cytosolic granules [33]. The PrLD of TIAR-2 was shown to be serine phosphorylated when expressed in mechanosensory neurons. Ten serine residues in the intrinsically disordered PrLD were predicted as phospho-sites using NetPhos3.1 [35]. Expression of a non-phosphorylatable (S→A) TIAR-2 mutant (at 10, 8, or 2 serine residues) showed significantly less granule formation in the axons of neurons when compared to wild type. Alternatively, phosphomimetic (S→E) TIAR-2 mutants showed similar levels of granule formation when compared to wild type. These data suggest phosphorylation of serine residues promotes LLPS and formation of TIAR-2 positive granules in neurons of *C. elegans* [33].

PTMs also affect virally encoded proteins and their LLPS capabilities in cells. Measles virus phosphoprotein is a 507-amino acid virally encoded protein composed of multiple IDRs. Measles virus phosphoprotein and nucleoprotein undergo LLPS to form IBs. Phosphoprotein is phosphorylated at multiple sites, but Serine 86 and Serine 151—both of which are in IDRs—have been identified as regulatory sites for IB formation. Mutation or inhibition of phosphorylation at these two sites results in irregular and small IBs [12].

Examples of serine/threonine phosphorylation that disrupt LLPS include maternal-effect germline proteins (MEGs) in P granules of *C. elegans* [36], fused in sarcoma (FUS) [37,38,39] and TAR DNA-binding protein 43 (TDP-43) [40]. Proper segregation of P granules in zygotes of *C. elegans* requires the expression of MEG proteins [41]. Interestingly, two of the MEG family proteins (MEG-1 and MEG-3) are phosphorylated within their IDRs by a regulatory kinase (MBK-2) [36]. MBK-2 activity and counteractive phosphatase (PPTR-1) activity on MEGs is required for P granule disassembly and formation, respectively [36].

FUS and TDP-43 are frequently studied proteins because their phase separation in vivo has been linked to amyotrophic lateral sclerosis (ALS) [42]. A current hypothesis is that MLOs containing these proteins may promote their stochastic conversion into solid, pathological aggregates [43]. FUS contains an intrinsically disordered N-terminal PrLD, which is necessary and sufficient to drive LLPS [5]. The ~160 amino acid PrLD has 32 putative phosphorylation sites, 12 of which have been identified as PIKK family kinase consensus sites [44]. Phosphomimetic substitution (S/T→E) at 6 or 12 PIKK consensus sites diminishes FUS’s ability to phase separate and form fibrillar aggregates in vitro [37]. In cells, a decrease in cytoplasmic aggregation is also observed upon increase in phosphomimetic substitution, suggesting a potential therapeutic target for disrupting pathological aggregate formation [37]. TDP-43 has a C-terminal PrLD, which is multiphosphorylated and aggregated in ALS motor neurons [45]. Two phosphorylation sites in the PrLD, Serine 409 and 410, identified in samples from frontotemporal lobar dementia patients were shown to regulate TDP-43 cytoplasmic granule formation. Phosphomimetic substitution (S→D) at Serine 409 and 410 showed a significant reduction in the number of cells containing TDP-43 puncta [46]. Interestingly, TDP-43 phase separation is regulated by PTMs in both an IDR and a structured domain. A single phosphorylation event in its N-terminal structured domain at Serine 48 is sufficient to suppress its LLPS in vitro and in cells [40]. Serine 48 is conserved in most species evaluated, including flies, mice, and humans [40].

There are two kinases, SKY1 and DYRK3, that have the ability to phase separate into SGs and, in the stages of recovery following a stress response, phosphorylate proteins containing IDRs, resulting in dissolution of the granules [47,48]. SKY1 is a yeast protein kinase with a PrLD that enables its recruitment into SGs. In SGs, SKY1 phosphorylates NLP3 at Serine 441, which is located in its serine-arginine rich C-terminal IDR [49]. This phosphorylation event promotes SG dissolution [48]. Similarly, DYRK3 (human homolog of MBK-2) was shown to phase separate into SGs via its intrinsically disordered N-terminal domain. Aside from regulation of SGs, DYRK3 was identified as a factor that controls phase separation and dissolution of several condensates containing IDPs during mitosis [50]. This kinase is interesting because it has broad-specificity and is generally proline-directed. Some proteins sensitive to DYRK3 inhibition, all of which contain IDRs, include splicing-speckle marker SC35, SG marker PABP, and pericentriolar-material protein PCM1. DYRK3 expression results in dissolution of these granules during mitosis, whereas a kinase-dead mutant or inhibition of DYRK3 results in the persistence of granules [50].

For some proteins, phosphorylation can be a driver or inhibitor of condensate formation, but there are instances where it is not clearly binary. Tau441 contains numerous IDRs and putative serine/threonine phosphorylation sites throughout the protein [51]. In experiments performed with bacterially produced recombinant full-length tau441 and molecular crowding agents, LLPS was driven mostly by electrostatic intermolecular interactions. There was no requirement for phosphorylation [52]. However, in a different study, phosphorylation was found to be required to initiate tau441 LLPS in vitro [34]. Of importance, there are 22 phospho-sites analyzed in this study, 15 of the sites are located in IDRs. Interestingly, LLPS of p-tau was dependent on hydrophobic interactions [34], whereas unphosphorylated tau LLPS was more dependent on ionic interactions [52]. These examples suggest the biophysical mechanism driving LLPS can be altered by the post-translational state of the protein.

### 3.2. Arginine Methylation

Methylation of arginine residues is important for regulating phase separation and recruitment of proteins into MLOs. The side chain of arginine contains a positively charged guanidinium head group that can be multiply methylated. This reaction is catalyzed by protein methyltransferases (PRMTs) using a donor methyl group from S-adenosyl methionine (SAM). Arginine methylation does not change the charge of the side group, but instead alters its volume, charge distribution, hydrophobicity, and potential for hydrogen bonding [53].

Including unmethylated arginine, there are four differential arginine methylation patterns. Arginines can be monomethylated (MMA) or dimethylated (DMA); arginine dimethylation can exist as symmetrical dimethylation (SDMA) or asymmetrical dimethylation (ADMA). Symmetric dimethylation occurs when two methyl groups are added to the two different nitrogen atoms within the guanidino group, whereas asymmetric dimethylation is when two methyl groups are added to the same nitrogen [54]. These reactions are catalyzed by different methyltransferases. PRMTs are grouped into three subtypes based on their catalytic activity. Type I PRMTs include PRMT1, 2, 3, 4, 6, and 8, while type II PRMTs include PRMT5 and 9 and there is one type III methyltransferase, PRMT7 [55,56]. All three classes of PRMTs have the ability to catalyze MMA reactions, but the DMA reactions that occur subsequent to this reaction are specific to type I and II enzymes. Type I PRMT enzymes catalyze the reaction of MMA to ADMA and type II enzymes catalyze MMA to SDMA. PRMT enzymes target arginine residues within glycine-arginine-glycine (GRG) or arginine-glycine-glycine (RGG) sequences [57], which are preferred sites of arginine methylation [58], and are frequently encoded as multiple repeats within low-complexity regions [6].

The hydrogen bond potential of the head group is the same for SDMA and ADMA, but the location of the methyl group can alter the orientation of hydrogen bonding. Additionally, SDMA and ADMA have different electrostatic surface potentials to the head group, resulting in shifting of charge [59]. The hydrophobicity of the head group is also modified upon methylation. Arginine hydrophobicity incrementally increases following the addition of MMA, ADMA, and SDMA, respectively [53]. Hydrophobic residues are generally located within the folded core of proteins, so within the context of an IDR, these changes may have profound effects on the propensity to fold or bind other macromolecules.

The guanidinium electrons are delocalized into π orbitals, enabling interactions via π-stacking [54]. Arginines can form cation-π interactions, which occur between the positively charged guanidinium group and the available electrons of the π orbital of aromatic rings [60]. These cation-π interactions have been shown to drive protein condensate formation [61]. Methylation of arginine has been shown to both favor and disfavor phase separation in certain contexts. ADMA in RGG motifs has been shown to disrupt favorable interactions and thus disrupt phase separation of IDPs such as dead-box helicase 4 (DDX4) [6], heterogeneous nuclear ribonucleoproteins A2 (hnRNPA2) [62], RAP55A [63,64], and FUS [65]. SDMA within RGG motifs, however, has been shown to drive phase separation. In the case of U6 snRNA-associated Sm-like protein (LSM4), SDMA is necessary for phase separation and processing body formation [66]. PRMT5 catalyzes SDMA of LSM4 at multiple arginine residues in its intrinsically disordered C-terminal RGG-containing domain. Mutation of the arginine residues in this domain or knockdown of PRMT5 diminish SDMA and processing body formation in cells [66].

YTH domain-containing family (YTHDF) of proteins contain numerous IDRs, which allows them to undergo LLPS. These proteins are found in cytoplasmic phase-separated SGs, P-bodies, and neuronal RNA granules [67]. In vitro experiments show that enzymatic modifications drive phase separation, but interestingly, the modifications occur to mRNA, not the protein. Methylation of adenosine in RNA, specifically the formation of N^6^-methyladenosine (m^6^A), is the most commonly modified nucleotide in mRNA. This modification increases mRNA’s multivalency and seeds phase separation of YTHDF proteins in vitro [67]. The protein has the ability to form droplets in solution without the presence of m^6^A, but only at much higher concentrations. Importantly, the mRNA that seeded droplet formation of YTHDF was multimethylated; unmethylated or singly methylated mRNA did not show droplet formation [67].

### 3.3. Arginine Citrullination

Citrullination is another PTM that occurs to arginine residues. Instead of the addition of a functional group, the arginine side chain undergoes an oxidation (or deimination) reaction. In this reaction, peptidylarginine deiminases (PADs) catalyze the oxidation of an imine group (=NH), forming a ketone group (=O) [60,68]. This modification removes the positive charge, leaving a neutrally charged amino acid. Interestingly, the consensus site for PADs are the same RG/RGG motifs that are common to many RNA-binding and phase separating proteins [69]. Citrullination of FUS by PAD4 was shown to diminish FUS recruitment to SGs [69]. PAD4 knockout in mouse embryonic fibroblasts showed a greater amount of FUS sequestration into SGs than when PAD4 was overexpressed in these cells, suggesting citrullination hinders FUS phase separation. Cation-π interactions between arginine residues and the π orbitals of tyrosine residues modulate FUS phase separation [60], but when citrullination occurs, the positive charge of the arginine side chain is removed, disrupting cation-π interactions and disrupting FUS phase separation in vitro [60].

### 3.4. Lysine Acetylation

Similar to citrullination, acetylation neutralizes the positive charge of an amino acid. Lysine residues contain a positively charged amino head group that can be neutralized by addition of an acetyl group; this not only changes lysine’s charge state but also increases its hydrophobicity [70]. Acetyl groups are enzymatically added via acetyltransferases and removed by deacetylases [71]. Acetylation has been shown to disrupt simple coacervation of DDX3X (dead box RNA helicase 3) in vitro [72]. DDX3X has two IDRs: at the C-terminus and the N-terminus. Analysis of an acetylome dataset identified several acetylated lysines in the N-terminal IDR that play a role in DDX3X incorporation into SGs [72]. DDX3X is a substrate of acetyltransferase CREB-binding protein (CBP) and histone deacetylase 6 (HDAC6). To better understand the role of acetylation of DDX3X and SG incorporation, acetyl mimetic (K→Q) constructs and acetyl-dead (K→R) were constructed and expressed in DDX3X knock-out cell lines. Expression of acetyl-dead DDX3X (or inhibition of CBP) increased SG volume, whereas expression of acetyl-mimetic mutant (or inhibition of HDAC6) decreased SG volume.

The formation of SG has been proposed as a two-step process. First a stable core structure is formed, which is followed by the recruitment of IDPs into an outer shell structure [73]. The increase in volume is an important step in SG maturation. Of significance to this growth mechanism, the interaction partners of the acetyl-dead and acetyl mimetic DDX3X mutants were different. The non-acetylated DDX3X interacts with numerous SG components, whereas the acetyl mimetic loses its capacity for interactions with SG proteins, thus showcasing how lysine acetylation can be used to regulate MLO maturation [72].

Another protein that is lysine acetylated is tau, which is of particular interest since its solid-phase aggregation in neurons is linked to Alzheimer’s disease [74]. Tau is an IDP, and like DDX3X, its ability to phase separate is disrupted by lysine acetylation [75]. Ferreon et al. found that recombinant tau, when incubated with enzymatically-active p300 histone acetyltransferase (HAT), becomes hyperacetylated (ac-tau) [75]. This acetylation (removal of positive charges) was observed to disfavor LLPS, which is consistent with the previous observations of Wegmann et al. that phosphorylation (addition of negative charges) promotes tau phase separation [34]. Using mass spectrometry analysis, 15 acetylation sites were identified, 8 of which are located in IDRs. Tau readily undergoes LLPS in vitro in low-salt conditions, but ac-tau was unable to form droplets under the same conditions. The neutralization of charged residues was concluded to disrupt electrostatic interactions required for tau LLPS [52,75]. Interestingly, tau also phase separates into SGs [76]. Ukmar-Godec et al. showed that tau association into SGs is altered by the acetylation state of the lysines. Unmodified full length tau441 readily associated with SGs following proteasome inhibition by MG132. Consistent with the in vitro findings above, acetylation of tau strongly reduced the association of the protein with SGs in HeLa cells [77]. Lastly, Ac-tau also showed decreased solid-phase aggregation propensity and reduced thioflavin-t reactivity, which indicates less propensity to form amyloid-like solid aggregates. This suggests acetyltransferases and deacetylases are potential therapeutic targets for prevention of pathological tau aggregation [75].

### 3.5. Poly(ADP-Ribosylation)

Poly(ADP-ribosyl)ation or PARylation is a reversible covalent addition of multiple NAD-derived ADP-ribose (ADPr) molecules to a protein [78]. ADPr units can be added to glutamate, aspartate, lysine, arginine, or serine residues by poly(ADP-ribose) polymerases (PARPs) and removed by PAR glycohydrolases (PARGs) [78]. Aside from the physical addition of ADPr units, polyADP-ribose (PAR) molecules are freely synthesized polymers that can modify phase separation of some IDPs [79]. PAR is a multivalent, anionic, nucleic acid-mimicking (similar to RNA) biopolymer that can be bound by phase separating proteins [2]. Cellular stress conditions and DNA damage have been shown to cause an upregulation of PAR synthesis [79]. PAR, PARPs, and PARGs have all been shown to play a regulatory role in SG dynamics [80].

The SG component hnRNPA1 contains both a PAR-binding domain and a PARylation site at Lysine 298 within a glycine rich IDR. PARylation at hnRNPA1 Lysine 298 is important for hnRNPA1 nucleocytoplasmic shuttling, a necessary step for localization to SGs following cellular stress [81]. Interestingly, like numerous other proteins, hnRNPA1 contains a PAR-binding motif (PBM). In vitro experiments showed hnRNPA1 phase separation increasing in response to increased PAR concentration in solution. Mutating the hnRNPA1 PBM resulted in no phase separation in the presence of PAR, implying this interaction is domain specific. TDP-43 also contains a PBM and co-phase separates with hnRNPA1 in vitro and in SGs [81,82]. In vitro, PAR binding via hnRNPA1 PBM is necessary for the co-phase separation of TDP-43 and hnRNPA1 in low-salt concentrations. In cells, both TDP-43 and hnRNPA1 need functional PBMs to localize to SGs, highlighting the role of PAR in protein–protein interaction and phase transition [81,82].

## 4. Membraneless Organelles and Neurodegenerative Diseases

A connection between MLOs and neurodegenerative disease has been widely observed [83]. Specifically, many proteins that are genetically or histopathologically linked to neurodegeneration are also found in neuronal MLOs [18]. Likewise, proteins with intrinsically disordered PrLDs are notoriously disproportionately linked to neurodegenerative disease [84], and many of these proteins are both capable of undergoing LLPS and frequently found within inclusions of diseased neurons [85].

Why do the same proteins that functionally undergo LLPS appear to adopt pathological meso-scale aggregates in cells? A leading hypothesis is proteins within MLOs may undergo additional transitions into oligomeric species, solid-phase aggregates [86], or droplet-like structures with dramatically different material properties [17] (Figure 1). For example, expression of an ALS-linked TDP-43 mutant results in an MLO that is more viscous and resistant to solvation, suggesting it has a stabilized internal structure [19]. Similarly, in vitro, ALS-mutant FUS can transition from a droplet state into a solid aggregate more rapidly than wild-type FUS [42]. Once formed, such aggregates are thought to be detrimental to cell function and contribute to neuronal degeneration. Possibly, the high concentration of specific proteins within MLOs may potentiate these stochastic, irreversible phase transitions. Since disease-linked proteins like tau and TDP-43 are hyper- and multi-phosphorylated, respectively, within neuronal cytoplasmic inclusions, it is possible the PTMs are facilitating solid-phase transitions; alternatively, the PTMs may simply mark failed attempts at solubilization.

In the case of many IDRs, the abundance of hydrophilic amino acids and lack of stable tertiary structures may facilitate solid-phase transitions into highly ordered amyloid conformations. Amyloid is a well-ordered, filamentous polymeric state composed of a single protein species, much like a one-dimensional crystal [87]. It usually consists of polypeptides aligning in parallel in-register beta sheets [88] and is notoriously difficult to solubilize. MLOs may provide an environment in which some enriched IDR-containing proteins can stochastically adopt amyloid-like conformations, thus explaining the presence of certain MLO-linked proteins in pathological neuronal inclusions. Examples include tau (Alzheimer’s disease), TDP-43 (ALS), and FUS (frontotemporal dementia). Importantly, crystal-like arrangements would be disrupted by PTMs occurring within the structural core of amyloid [89]; thus, targeting specific modifying enzymes could offer a viable therapeutic strategy for neurodegenerative disorders that feature solid-phase inclusions.

## 5. Future Directions

Experimentation with MLOs is frequently focused on a few protein species. However, in vivo, the entire repertoire of macromolecules within individual biocondensates remains largely unknown. Additionally, for any given MLO, specific protein components can exhibit a broad array of PTMs, thus making it difficult to dissect which modifications are altering LLPS or perhaps serving other non-structural functional roles. Going forward, a major challenge will be to determine the precise relationship between MLOs and disease processes. For example, many human viruses encode proteins with PrLDs [90]. Given what we know about this type of protein domain, it is possible many viruses exploit LLPS during replication and infection, yet antiviral drugs do not specifically target LLPS mechanisms. In the case of neurodegenerative diseases, the pathological connections between aberrant phase transitions and neuronal death are not fully understood, and there are many non-unifying hypotheses. No drugs specifically target phase-separation processes in any neurodegenerative disease. Yet, given the almost complete lack of drugs for treating these diseases, manipulating the enzymes that regulate biocondensation may provide a new target paradigm.

## Figures and Tables

**Table 1:** Examples of post-translational modifications (PTMs) of intrinsically disordered regions (IDRs) altering the liquid–liquid phase separation (LLPS) of proteins. The underlined proteins have multiple PTMs that affect the phase separation propensity. Arrows indicate if PTMs promote (↑) or inhibit (↓) LLPS. C-terminal domain (CTD), N-terminal domain (NTD), prion-like domain (PrLD), arginine-glycine-glycine (RGG), stress granules (SGs), amyotrophic lateral sclerosis (ALS), and frontal temporal dementia (FTD).

PTM	Protein Example	Region Modified	Proposed Effects of PTM on LLPS (↓↑)		Type of MLO	Disease Link
Serine/Threonine Phosphorylation	FMRP	CTD IDR	↑	Increases electrostatic interactions	Neuronal granules	Fragile X syndrome
	TIAR-2	CTD PrLD	↑		SGs	
	Phosphoprotein	Internal IDR	↑		Inclusion bodies	Measles
	tau	Internal IDR	↑		SGs	Alzheimer’s disease
	MEG-3	Internal IDR	↓	Introduces electrostatic repulsion	P granule	
	FUS	NTD PrLD	↓		SGs, nuclear paraspeckles	ALS, FTD
	TDP-43	NTD domain & CTD IDR	↓		SGs	ALS, FTD
Arginine Methylation	(SDMA) LSM4	49 aa, NTD RGG domain	↑	Changes hydrophobicity and H-bonding	Processing bodies	
	(ADMA) hnRNPA2	CTD IDR at RGG sites	↓		SGs	ALS, FTD
	RAP55A	36 aa, NTD RGG domain	↓	Changes hydrophobicity and H-bonding	SGs, Processing bodies	Primary biliary cirrhosis
	FUS	41 aa, CTD RGG domain	↓		SGs, nuclear paraspeckles	ALS, FTD
Arginine Citrullination	FUS	RGG domain	↓	Disrupts charge-charge interactions	SGs, nuclear paraspeckles	ALS, FTD
Lysine Acetylation	DDX3X	NTD IDR	↓	Disrupts cation–π interactions	SGs	Intellectual disability
	tau	Internal IDR	↓		SGs	Alzheimer’s disease
Lysine Ribosylation	hnRNPA1	Glycine-rich region	↑	Increases multivalency	SGs	ALS, FTD

## Abbreviations

ADMA	Asymmetric dimethyl arginine
ADPr	NAD-derived ADP-ribose
IBs	Inclusion bodies
IDP	Intrinsically disordered protein
IDR	Intrinsically disordered region
LCD	Low-complexity domain
LLPS	Liquid–liquid phase separation
MMA	Monomethyl arginine
MLO	Membrane-less organelle
PTM	Post-translational modification
PrLD	Prion-like domain
SDMA	Symmetric dimethyl arginine
SG	Stress granule
RGG/RGR	Arginine/glycine repeats
